# Echocardiographic Markers of Right Ventricle Diastolic Dysfunction in Neonates and Infants with Congenital Heart Disease

**DOI:** 10.3390/jcm15010098

**Published:** 2025-12-23

**Authors:** Massimiliano Cantinotti, Guglielmo Capponi, Marco Scalese, Eleonora Palladino, Raffaele Giordano, Eliana Franchi, Cecilia Viacava, Giulia Corana, Pietro Marchese, Alessandra Pizzuto, Nadia Assanta, Giuseppe Santoro

**Affiliations:** 1 Fondazione CNR-Regione Toscana G. Monasterio, 54100 Massa, Italy; cantinotti@ftgm.it (M.C.); gcapponi@ftgm.it (G.C.); elepalla06@gmail.com (E.P.); efranchi@ftgm.it (E.F.); cviacava@ftgm.it (C.V.); corana@ftgm.it (G.C.); pmarchese@ftgm.it (P.M.); apizzuto@ftgm.it (A.P.); assanta@ftgm.it (N.A.); 2 Institute of Clinical Physiology, 56124 Pisa, Italy; scalese@ifc.cnr.it; 3 Adult and Pediatric Cardiac Surgery, Department of Advanced Biomedical Sciences, University of Naples “Federico II”, 80131 Napoli, Italy; r.giordano81@libero.it

**Keywords:** echocardiography, children, neonates and infants, right ventricle, diastolic dysfunction

## Abstract

**Background:** Assessing right ventricular (RV) diastolic function by echocardiography in pediatric patients remains complex, particularly in congenital heart disease (CHD) characterized by RV pressure overload. The geometric peculiarities of the RV, respiratory influences, and age-dependent maturational changes complicate interpretation of Doppler-derived indices. This study aimed to characterize tricuspid valve (TV) pulsed-wave Doppler E/A inflow patterns in infants with CHD and RV pressure overload, evaluated shortly after surgical or percutaneous intervention. **Methods:** Echocardiographic analysis included TV E- and A-wave velocities obtained by pulsed-wave Doppler and measurement of E-wave deceleration time (EDT). Beat-to-beat variability was quantified over three consecutive cardiac cycles. Data were compared with a large cohort of age-matched healthy children. **Results:** Fifty-seven infants with CHD (35 pulmonary stenosis; 22 tetralogy of Fallot), examined 12–48 h post-intervention, were compared with 134 healthy controls. CHD patients showed markedly reduced beat-to-beat variability of both E- and A-wave velocities (*p* < 0.001 and *p* = 0.007, respectively). A three-beat E/A inversion pattern—common in neonates but variable in healthy infants—was consistently observed in CHD patients (*p* < 0.001). A-wave velocities were significantly higher (*p* < 0.001), whereas E/A ratios (*p* < 0.001) and EDT values (*p* = 0.010) were significantly lower compared with controls. **Conclusions:** Infants with CHD and RV pressure overload exhibit a characteristic Doppler pattern consisting of E/A ratio inversion, reduced beat-to-beat variability, increased A-wave velocity, and shortened E/A ratio and EDT. These findings may serve as practical and reproducible indicators of RV diastolic dysfunction in the early post-intervention period in neonates and infants.

## 1. Introduction

Evaluating right ventricular (RV) diastolic function in pediatric patients is inherently challenging due to the complex morphology of the RV, its heightened sensitivity to respiratory fluctuations, and the rapid maturation of myocardial compliance throughout infancy and childhood [1,2,3,4,5,6]. These developmental factors, combined with substantial heterogeneity in congenital and acquired heart disease, limit the applicability of adult diagnostic paradigms and reinforce the need for pediatric-specific criteria. Nevertheless, many clinical decisions continue to rely on adult-derived frameworks [7,8] because comprehensive pediatric datasets remain limited. Existing studies often include small cohorts, focus on isolated disease mechanisms such as pulmonary hypertension [1,2,3,4,5], and frequently lack uniform methodology [9,10,11,12]. Consequently, the interpretation of right-sided diastolic indices in childhood must account for both age-specific physiology and disease-related variations in loading conditions.

Recent pediatric Doppler nomograms [9] have clarified the significant age-related evolution of tricuspid inflow patterns, particularly the E/A ratio. In contrast to adults, where E < A typically indicates impaired relaxation [7,8], neonates and young infants commonly exhibit physiological E/A inversion. This reflects developmental characteristics such as reduced early diastolic suction, higher myocardial stiffness, and prominent dependence on atrial contraction. Indeed, three-beat E/A inversion has been documented in most healthy neonates (71%) and remains evident during the first two years of life, with gradual transition toward adult-like E > A patterns thereafter [9]. Even beyond infancy, sporadic E/A inversion persists in otherwise healthy children, highlighting the variability intrinsic to pediatric physiology.

Beyond the context of pulmonary hypertension, several congenital heart diseases (CHD) featuring RV pressure overload—including tetralogy of Fallot (TOF) and pulmonary valve stenosis (PS)—may further modify RV relaxation and compliance. These alterations often become most apparent immediately following surgical repair or catheter-based intervention, when abrupt shifts in preload, afterload, and ventricular interaction occur. Such dynamic changes create a unique opportunity to study RV diastolic behavior under conditions of both structural abnormality and rapidly evolving hemodynamics.

Within this framework, the present study sought to characterize tricuspid E/A inflow patterns in infants with CHD associated with RV pressure overload. Specific attention was given to the early postoperative or post-interventional period, a phase during which transient physiological perturbations may unmask subtle abnormalities in diastolic function. By comparing these patients to a large cohort of well-phenotyped healthy children, the study aimed to identify distinctive Doppler features that may serve as clinically meaningful indicators of early diastolic dysfunction in this population.

## 2. Methods

### 2.1. Study Population

#### 2.1.1. Children with CHD

Children with congenital heart disease (CHD) associated with right ventricular (RV) pressure overload were prospectively enrolled between June 2021 and June 2025 at a single tertiary-level institution. All patients underwent either biventricular surgical repair or catheter-based intervention according to standard clinical practice. Echocardiographic assessment was performed in a uniform temporal window, between 12 and 48 h after the procedure, to ensure a consistent early post-interventional evaluation across the cohort.

#### 2.1.2. Exclusion Criteria

To minimize sources of variability in tricuspid inflow analysis, several exclusion criteria were applied. Children with tricuspid valve stenosis or more than moderate tricuspid regurgitation were not included, as these conditions directly affect Doppler-derived inflow parameters. In addition, patients who were not breathing spontaneously at the time of imaging were excluded, as were those receiving inotropic support, in whom altered loading conditions could significantly influence diastolic measurements. To maintain population homogeneity, syndromic patients and those with known chromosomal abnormalities were excluded from final analysis.

### 2.2. Healthy Controls

The control cohort consisted of 134 healthy children selected by age-matching from a previously validated normative dataset comprising 554 subjects. The controls had a mean age of 3.42 ± 4.63 months, a median age of 2.95 months, and an interquartile range of 0.9–5.8 months. Of these children, 65 were female, corresponding to 48.5% of the sample. All controls underwent echocardiography for routine screening or non-cardiac indications and displayed structurally normal hearts.

Children with congenital or acquired heart disease were excluded. Minor anatomical variants such as a patent foramen ovale were considered within normal limits. Additional exclusion criteria encompassed any known or suspected neuromuscular disorder, genetic syndrome, or chromosomal abnormality; anthropometric measurements indicative of obesity according to World Health Organization growth standards; a diagnosis of pulmonary hypertension; systemic hypertension in children older than four years; a history of connective tissue disease; or a family history of inherited cardiac disorders, including cardiomyopathies or connective tissue disorders such as Marfan syndrome. These criteria ensured that the control population represented physiologically normal tricuspid inflow across the age spectrum included in the study.

### 2.3. Echocardiographic Examinations

All echocardiographic studies were performed using comprehensive two-dimensional imaging, with digital acquisition of images for subsequent offline analysis. Examinations were conducted only in calm, cooperative children, without sedation, in order to avoid artificial modifications of respiratory mechanics or hemodynamic status. Standard apical four-chamber views were used to obtain pulsed-wave Doppler tracings of tricuspid valve inflow. The parameters recorded included early diastolic (E-wave) and late diastolic (A-wave) peak velocities and the E-wave deceleration time (EDT). Each parameter was derived by averaging measurements obtained over three consecutive cardiac cycles to account for physiological respiratory variability.

Left ventricular ejection fraction (LVEF) was calculated using the biplane Simpson method. Global longitudinal strain (GLS) was measured for both ventricles, including left ventricular GLS (GLVLS) and right ventricular GLS (GRVLS), following previously standardized methodologies. Right ventricular systolic pressure was estimated from the peak gradient obtained from tricuspid regurgitant velocity, and fractional area change (FAC) was determined by measuring end-diastolic and end-systolic RV areas in the four-chamber view. Only studies with high-quality images and clearly defined Doppler envelopes were included in the analysis.

To ensure methodological consistency, two experienced pediatric cardiologists (M.C. and E.F.) independently acquired and evaluated all imaging data. Both operators followed the same institutional protocol, thereby minimizing operator-dependent variability.

### 2.4. Reproducibility of Tricuspid Doppler Measurements

To evaluate the reliability of tricuspid Doppler measurements, inter-operator and intra-operator reproducibility were assessed in a group of 20 randomly selected CHD patients. Intra-operator reproducibility was evaluated by repeating measurements after a one-week interval, while inter-operator reproducibility was determined by comparing values acquired independently by the two cardiologists on the same datasets. The coefficient of variation (CV) for each parameter was calculated as the means of individual CVs from duplicate measurements. A CV of less than 15% for inter-operator comparisons and less than 10% for intra-operator comparisons was considered acceptable. Intraclass correlation coefficients (ICC) were also computed to quantify the degree of agreement between repeated measurements

### 2.5. Statistical Analysis

Continuous variables were expressed as mean ± standard deviation (SD) when normally distributed or as median and interquartile range (IQR) when distribution was skewed. Categorical data were summarized as absolute counts and percentages. Comparisons between groups were performed using the chi-squared test for categorical variables. For continuous variables, Student’s *t*-test or ANOVA was used when data were normally distributed, whereas Mann–Whitney U and Kruskal–Wallis tests were used when normality assumptions were not met. When multiple comparisons were required, Bonferroni correction was applied to adjust for inflated type I error risk.

Beat-to-beat variability in E- and A-wave velocities and EDT was quantified by computing the standard deviation across three consecutive cardiac cycles, allowing differentiation between fixed and physiologically variable inflow patterns. All analyses were carried out using SPSS, Release 23.0 (IBM, Chicago, IL, USA), and Stata, Version 13 (Stata Corporation, College Station, TX, USA). A two-tailed *p* value < 0.05 was considered statistically significant.

### 2.6. Ethical Approval and Informed Consent

The study received formal approval from the Local Ethics Committee (Study “Bet,” N.390). Written informed consent was obtained from all parents or legal guardians after they were thoroughly informed about the study objectives and procedures.

## 3. Results

### 3.1. Study Population

Of the 149 subjects initially evaluated, 92 were excluded because they were not breathing spontaneously (n = 77, including 40 receiving inotropic support) or due to incomplete or suboptimal echocardiographic studies (n = 15). The final study cohort consisted of 57 children aged 0–24 months, including 22 with TOF and 35 with PS. Echocardiography was performed 12–36 h after the intervention. All infants were hemodynamically stable and breathing spontaneously during acquisition. Thirty-five children required nasal oxygen at 0.5–1.0 L/min, while none required inotropic support at the time of the study. All patients with PS and ten with TOF had a residual PFO. In all cases the shunt was predominantly left-to-right, except for three neonates who presented a truly bidirectional flow (Table 1).

### 3.2. Feasibility

Not all parameters were obtainable in every patient, although overall feasibility remained good. In health controls, acquisition success ranged from 98.0% to 99.3%, reflecting optimal imaging conditions. In children with CHD, feasibility ranged from 84.8% to 90.6%, likely reflecting postoperative variability or transient movement. Despite these limitations, most Doppler measurements could be analyzed, enabling adequate assessment of early postoperative RV diastolic patterns.

### 3.3. Patterns of TV Inflow in Children with CHD

In the CHD cohort, 32 patients were neonates aged 3–31 days (all with PS), whereas 25 were infants aged 1–24 months (22 with TOF and 3 with PS). Across the entire group, three-beat E/A inversion was significantly more frequent in CHD patients than in controls (*p* < 0.001). When neonates were evaluated separately, the difference was not statistically significant (93.8% in CHD vs. 80% in healthy neonates; *p* = 0.363) (Table 2).

**Figure 1:**
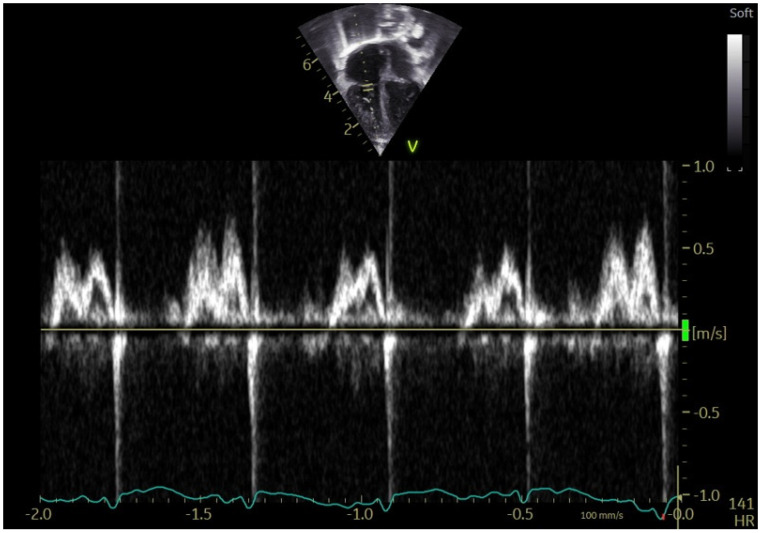
Physiological TV E/A variability in a healthy infant 3 months of age.

**Figure 2:**
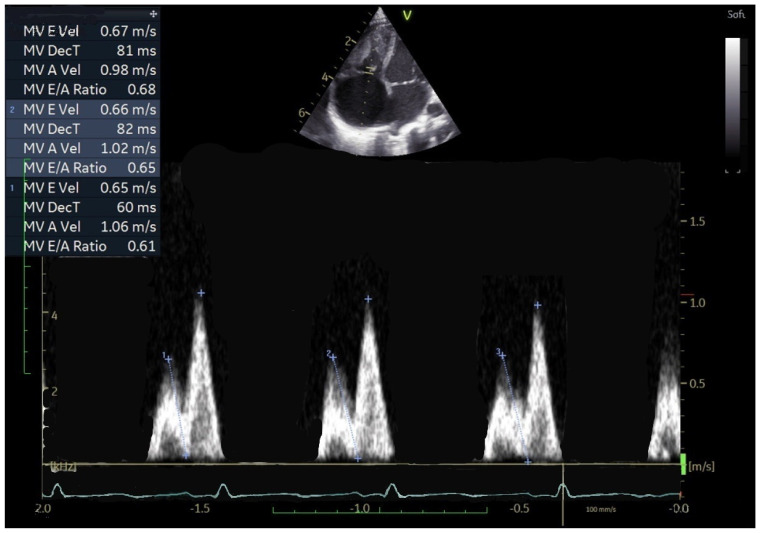
Fixed E/A pattern in a newborn, 7 days old, 3 Kg of weight, with pulmonary stenosis after percutaneous valvuloplasty.

The tricuspid inflow pattern in CHD patients showed marked stability, with significantly reduced beat-to-beat variability in E-wave velocity (*p* < 0.001) and A-wave velocity (*p* = 0.007). A-wave velocities were significantly increased (*p* < 0.001), while both the E/A ratio (*p* < 0.001) and EDT (*p* = 0.010) were reduced compared with healthy children. These characteristics are illustrated in Figure 1 and Figure 2.

### 3.4. Correlations

Correlation analysis between TV Doppler variables (E and A wave velocities, E/A ratio, EDT, and three-beat inversion) and ventricular performance indices (LV EF, LV GLS, RV GLS, RV free-wall GLS, RV FAC, estimated RV systolic pressure, and oxygen saturation) identified only two significant associations. A-wave velocity showed a positive correlation with estimated RV pressure (r = 0.443, *p* = 0.013) and with LV GLS (r = 0.447, *p* = 0.042). EDT correlated positively with respiratory rate (r = 0.381, *p* = 0.017), aligned with its dependence on respiratory mechanics

### 3.5. Confounders

No significant differences were observed between infants who received nasal oxygen supplementation and those breathing room air. Similarly, the presence of a PFO or a residual shunt did not influence tricuspid Doppler parameters. The three neonates with truly bidirectional PFO flow did not differ significantly, although interpretation is limited due to the small number of cases.

### 3.6. Reproducibility of Tricuspid Doppler Measurements

Reproducibility was excellent for all principal Doppler variables. Intraclass correlation coefficients (ICC) for intra-operator measurements ranged from 0.95 to 0.98, and inter-operator ICC values ranged from 0.93 to 0.97, confirming high reliability for E-wave velocity, A-wave velocity, E/A ratio, and EDT.

## 4. Discussion

This study provides a detailed analysis of right ventricular diastolic function in neonates and infants with CHD associated with RV pressure overload, specifically focusing on TOF and PS, during the early post-intervention period. Pediatric RV physiology differs substantially from adults due to unique myocardial compliance, ventricular geometry, and rapid age-related changes in preload and afterload. These characteristics complicate the direct application of adult-based E/A ratio thresholds for assessing diastolic dysfunction [6,7,8,13,14].

Indirect echocardiographic markers such as right atrial enlargement, leftward septal displacement, right-to-left shunting via PFO, and systemic venous congestion (pleural effusion, ascites) are commonly used to infer RV filling pressures [6,10,11,12]. Although informative, these markers do not directly quantify RV contribution to diastolic filling and may fail to detect subtle alterations, particularly in the early postoperative period when hemodynamics are dynamic.

Previous studies, including our normative work, have shown that physiologic E/A inversion is common in neonates and infants [9,15,16,17,18]. Sustained inversion across three consecutive beats is observed in up to 70% of healthy neonates and infants [9,15,16,17,18]. These findings indicate that three-beat inversion alone is insufficient to reliably identify diastolic dysfunction in this age group. Age-related changes in E and A velocities and in E-wave deceleration time further complicate the use of fixed diagnostic thresholds [9,15,16,17,18].

In the present CHD cohort, infants exhibited markedly reduced beat-to-beat variability in E and A velocities, nearly fixed three-beat E/A inversion, increased A-wave velocities, and shortened E-wave deceleration time. This pattern reflects impaired RV relaxation with augmented atrial contribution to ventricular filling, consistent with early diastolic dysfunction. While three-beat inversion can be physiologic in neonates, its persistent presence along with minimal variability in CHD patients suggests that the stability of inflow, rather than inversion itself, is a more sensitive indicator of impaired relaxation.

Physiologically, reduced beat-to-beat variability likely reflects diminished RV capacity to adapt early diastolic filling to small changes in preload. In healthy neonates [9,16,17,18], high myocardial compliance and respiratory-driven preload fluctuations generate natural variability in E- and A-wave velocities. Pressure overload increases myocardial stiffness and delays relaxation, limiting the physiological modulation of inflow velocities by respiration [1,2,12]. Consequently, Doppler patterns become “fixed,” with restricted dynamic adaptation, highlighting impaired lusitropy and a greater reliance on atrial contraction [19,20]. This explains the consistent E/A pattern observed in CHD patients despite variable loading conditions.

Individual neonatal examples further illustrate early dysfunction. One six-day-old infant with PS after valvuloplasty showed nearly equal E and A velocities without inversion, but minimal beat-to-beat variability and increased A-wave contribution. Another neonate demonstrated inversion in only two beats yet similarly had restricted variability and elevated A-wave velocity. These cases indicate that reduced physiological variability may serve as an early and sensitive marker of RV diastolic impairment even when classic inversion criteria are not fully met.

Early postoperative changes in RV loading conditions likely enhance these patterns. Surgical or catheter-based relief of obstruction abruptly modifies preload and afterload, affecting tricuspid inflow and potentially exaggerating the fixed E/A pattern. Therefore, both intrinsic myocardial relaxation deficits and transient hemodynamic effects contribute to early postoperative diastolic abnormalities.

Clinically, these findings suggest several implications. Adult-derived diastolic indices [7,8], such as E/A inversion, should not be interpreted in isolation in pediatric populations [9,13]. Integrating E/A inversion with beat-to-beat variability and A-wave magnitude offers a more physiologically appropriate assessment of RV diastolic function. This combination may provide a reproducible, non-invasive tool for postoperative monitoring. Moreover, the study underscores the need for age-specific reference values for tricuspid inflow. Establishing normative data across pediatric ages would improve differentiation between physiological variability and early pathological changes. Diastolic assessment should also be contextualized with global RV mechanics, including longitudinal strain, FAC, and RV systolic pressure, which collectively influence filling [19,20].

In summary, the results support the use of tricuspid E/A inflow patterns and beat-to-beat variability as indicators of RV diastolic function in infants with CHD. This approach enhances understanding of pediatric RV physiology in the early postoperative period and highlights the importance of age-appropriate interpretation of Doppler parameters. Rapid relief of obstruction and transient preload/afterload changes may accentuate fixed E/A inversion and reduced variability, reflecting both intrinsic relaxation deficits and hemodynamic influences. These reproducible markers can guide postoperative monitoring and identify patients at risk of delayed RV recovery or adverse outcomes.

## 5. Limitations

Complete echocardiographic datasets were not available for all participants. Although tissue Doppler imaging (TDI) is recognized as a useful tool for evaluating RV diastolic function, it was excluded from the analysis due to limited feasibility (<70%) in neonates and infants, primarily because of high heart rates, restricted cooperation, and technical difficulties. Advanced parameters such as right atrial (RA) strain, while promising in older children and adults with pulmonary hypertension [3,5,19,20], also show limited reproducibility in the youngest patients. Additionally, the cohort consisted exclusively of patients with TOF and PS, limiting the generalizability of findings to other CHD types. Early postoperative assessment may also be influenced by transient hemodynamic fluctuations and surgical stress. Importantly, all patients included were hemodynamically stable, spontaneously breathing, and without respiratory support, which minimized respiratory phase effects on tricuspid E/A parameters. Comparisons with intubated children or those on invasive respiratory support have shown that E/A patterns do not differ substantially from the selected cohort, suggesting applicability to the early postoperative population. All results can be interpreted only at the population level. At the individual level, no definite physiological significance can be inferred for a given subject because the mean absolute values of all parameters were very similar between healthy and CHD groups, with substantial overlap in standard deviations and no identifiable cut-off value for any parameter. Moreover, in this population, the challenge of normalizing atrioventricular Doppler parameters for age and body size further limits the feasibility of constructing and applying z-scores with adequate statistical power [9].

### Future Perspective

Speckle-tracking echocardiography (STE) for atrial strain assessment may enhance feasibility and reproducibility even in neonates and infants with high heart rates. Future evaluations of RV diastolic function in pediatric CHD should integrate conventional Doppler markers with advanced echocardiographic techniques to improve diagnostic sensitivity and prognostic assessment. Atrial STE provides quantitative data on reservoir and conduit function, refining the assessment of atrioventricular interactions [21,22,23].

Right atrial mechanics are increasingly recognized as key determinants of RV preload in conditions of RV pressure overload. Reduced RA reservoir strain correlates with elevated RA pressures and predicts adverse outcomes more accurately than conventional parameters in pulmonary hypertension (r = −0.69, *p* < 0.001; 20.3 ± 5.6% vs. 34.1 ± 6.7%, *p* < 0.001) [21,24,25]. Including RA strain [21,22,23,24,25] alongside Doppler indices may therefore provide a more complete evaluation of early RV diastolic dysfunction in neonates and infants, particularly in the immediate postoperative period.

Blood speckle-tracking (BST) echocardiography also shows potential for estimating intraventricular pressure differences (IVPD) during early diastole [26]. IVPD is significantly lower in children with CHD [27] and in cardiomyopathies [26], indicating reduced diastolic suction and altered filling dynamics [26,27].

A multistep diagnostic algorithm combining conventional Doppler measurements, beat-to-beat variability, atrial strain, RV longitudinal mechanics [21,22,23,24,25], and BST-derived IVPD [26,27] could improve early detection of diastolic dysfunction and support risk stratification for delayed RV recovery or long-term impairment.

## 6. Conclusions

In this study, Doppler-derived tricuspid valve E/A inflow patterns were evaluated in children with congenital heart disease characterized by right ventricular pressure overload and established RV diastolic dysfunction. The findings demonstrate that this CHD population consistently exhibits an inverted E/A ratio, a fixed inflow pattern with minimal beat-to-beat variability, elevated A-wave velocities, and reduced E/A ratio values. These echocardiographic features appear to provide simple, rapid, and reproducible markers of RV diastolic dysfunction in neonates and infants.

## Figures and Tables

**Table 1:** Patients characteristics.

	*All*	*3–31 Days CHD*	*1–24 Months CHD*
***Age (months)***	3.37 ± 4.71	0.27 ± 0.18	7.34 ± 4.75
***Weight (kg)***	4.76 ± 2.27	3.21 ± 0.47	6.84 ± 2.05
***Height (cm)***	56.6 ± 9.9	50.3 ± 2.9	65.1 ± 9.5
***BSA (m^2^)***	0.27 ± 0.09	0.21 ± 0.02	0.36 ± 0.08
***TOF***	22	0	22
***PS***	35	32	3
***RR (a.p.m.)***	40.67 ± 11.25	39.84 ± 8.59	41.94 ± 14.64
***HR max (b.p.m.)***	151.21 ± 161.59	162.85 ± 191.37	128.72 ± 14.40
***HR min (b.p.m.)***	129.51 ± 15.20	130.1 ± 15.97	122.63 ± 11.40
***Oxygen saturation%***	94 ± 8.1	92.1 ± 9.5	96.9 ± 4.1
***LV GLS %***	−18.74 ± 3.74	−18.87 ± 3.69	−18.62 ± 3.95
***LV EF %***	59.73 ± 5.42	60.08 ± 4.71	59.09 ± 6.70
***RV GLS %***	−16.42 ± 6.87	−16.18 ± 8.50	−16.66 ± 5.17
***RV GLS free wall %***	−14.10 ± 4.65	−13.18 ± 5.22	−15.31 ± 3.86
***Estimated RV pressure (mmHg)***	47.4 ± 17.5	50 ± 17.7	40.4 ± 16.1

a.p.m. = acts per minute, BSA = body surface area, b.p.m. = beats per minute, HR = heart rate LV = left ventricle, GLS = global longitudinal strain, PS = pulmonary stenosis, RV = right ventricle, RR = respiratory rate, TOF = tetralogy of Fallot.

**Table 2:** TV E/A pattern in healthy subjects and in those with CHD characterized by RV pressure overload.

	*0–31 Days* *Healthy*	*0–31 Days* *CHD*	*p*	*1–24 Months* *Healthy*	*1–24 Months CHD*	*p*	*0–24 Months* *Healthy*	*0–24 Months* *CHD*	*p*
***E veloc*** ***cm/s***	0.58 ± 0.39	0.59 ± 0.19	0.221	0.60 ± 0.14	0.68 ± 0.20	0.101	0.60 ± 0.22	0.63 ± 0.19	0.382
***A vel*** ***cm/s***	0.62 ± 0.13	0.92 ± 0.28	<0.001	0.61 ± 0.16	0.78 ± 0.26	0.02	0.61 ± 0.15	0.87 ± 0.28	<0.001
***E DT ms***	86.7 ± 29.1	79.6 ± 27.2	0.364	97.8 ± 31.5	87.5 ± 21.4	0.161	95.5 ± 31.2	82.7 ± 25.1	0.010
***E/A***	1.12 ± 1.50	0.66 ± 0.15	0.004	1.08 ± 0.42	0.88 ± 0.18	0.128	1.09 ± 0.79	0.75 ± 0.20	<0.001
***E vel sd***	0.04 ± 0.04	0.03 ± 0.03	0.120	0.07 ± 0.06	0.03 ± 0.02	<0.001	0.07 ± 0.06	0.03 ± 0.03	<0.001
***A vel sd***	0.05 ± 0.04	0.06 ± 0.07	0.511	0.07 ± 0.05	0.04 ± 0.02	0.006	0.07 ± 0.05	0.05 ± 0.06	0.007
***E/A no inversion***	3 (12%)	1 (3.1%)	0.363	26 (30.2%)	5 (23.8%)	0.029	29 (26.1%)	6 (11.3%)	<0.001
***E/A inversion 1 beat***	1 (4%)	0 (0%)		13 (15.1%)	0 (0%)		14 (12.6%)	0 (0%)	
***E/A inversion 2 beats***	1 (4%)	1 (3.1%)		9 (10.5%)	0 (0%)		10 (9%)	1 (1.9%)	
***E/A inversion 3 beats***	20 (8%)	30 (93.8%)	0.363	38 (44.2%)	16 (76.2%)	0.029	58 (52.3%)	46 (86.8%)	<0.001

CHD = congenital heart disease, E/A = E velocity/A velocity ratio, EDT = E wave deceleration time, vel = velocity, sd = standard deviation.

## Data Availability

The data presented in this study is available on request from the corresponding author. The data is not publicly available due to privacy issues.
